# Comparison of recording of pericarditis and lung disorders at routine meat inspection with findings at systematic health monitoring in Danish finisher pigs

**DOI:** 10.1186/s13028-015-0109-z

**Published:** 2015-03-29

**Authors:** Søren S Nielsen, Gitte B Nielsen, Matthew J Denwood, John Haugegaard, Hans Houe

**Affiliations:** Department of Large Animal Sciences, Faculty of Health and Medical Sciences, University of Copenhagen, Grønnegårdsvej 8, DK-1870 Frederiksberg C, Denmark; MSD Animal Health, Lautrupbjerg 4, DK-2750 Ballerup, Denmark

**Keywords:** Abattoir data, Diagnostic evaluation, Meat inspection recordings, Pericarditis, Pleuritis, Slaughter pigs

## Abstract

**Background:**

The use of secondary data is widespread in a range of surveillance and monitoring applications because of the low cost and high availability associated with this form of data. However, as they are often collected for quite unrelated purposes, they are not necessarily fit for the new purpose that is required of them. Routine meat inspection data were originally collected with the purpose of safeguarding food, but have been re-tasked to also include animal welfare assessment. The objective of the present study was to compare the recording of pericarditis, pleuritis and lungs with lesions at routine meat inspection (RMI) with those performed at systematic health monitoring (SHM) in Danish pigs at slaughter, in order to assess the usefulness of RMI for monitoring the prevalence of these diseases. Data originating from 165 Danish pig herds were collected in the period September 2011 to November 2013. From each herd, a batch consisting of all pigs slaughtered on a specific day from a specific farm were included as the RMI data, while lungs and hearts sampled from the batches were used for the SHM. The RMI data and SHM data included recordings related to a) chronic pericarditis, b) chronic pleuritis and c) lung lesions. The proportion of carcases with a specific disease recording was estimated for each batch of pigs, and linear regression was used to relate the RMI-proportion to the SHM-proportion for the conditions mentioned above.

**Results:**

The coefficients of determination (R^2^) were estimated as R^2^_,pericarditis_ = 0.16; R^2^_,pleuritis_ = 0.67; R^2^_,lungs with lesions_ = 0.40. R^2^_,pericarditis_ changed to 0.42 when the regression analysis included inspection type at the abattoir (with purely visual inspection of the hearts versus traditional inspection including an incision into the heart).

**Conclusions:**

Overall, the results suggest that the correlation between findings at RMI and SHM was moderate for pleuritis and lungs with lesions, but poor for pericarditis. The latter could partly be explained by the type of meat inspection conducted at the abattoir. We conclude that caution should be used whenever RMI data are used for purposes other than those for which they were originally intended.

## Background

Collection of data from production animals is done for a variety of purposes such as; to increase food safety or animal health; for decision making in animal production; and to ensure animal welfare. For example, all pig carcasses are subject to routine meat inspection (RMI) according to EU and Danish legislation [1,2] for the purposes of safeguarding food and animal welfare at slaughter. However, the resulting data may also be used for other purposes such as generating basic prevalence estimates for specific conditions in herd health advisory services. Whereas meat inspection was originally introduced to find food not suitable for human consumption (c.f.[3]), there is a drive to use these ‘cheap’ secondary data source for purposes other than those for which they were originally intended; for example animal welfare control and assessments [4,5]. However, these data may not provide useful estimates of herd health because they may not meet the fitness-for-purpose criterion. In addition, the threshold for recording of abnormalities may be related to different purposes, but may also be related to variation among observers.

In Denmark, systematic health monitoring (SHM) of lung and heart is an option for farmers having clinical problems with e.g. pneumonia, i.e. the diagnostic purpose relates to animal health [6,7]. Christensen and Enøe [7] noted that the prevalence of pleuritis and pneumonia recorded at RMI and SHM differed to some extent, and that agreement beyond that expected by chance (expressed as Kappa-values) between RMI and SHM were in the range 0.29 and 0.64. Furthermore, the sensitivity and specificity of RMI is not perfect, e.g. Bonde *et al.* [8] estimated that detection of heart related conditions had an apparent “sensitivity” of 49% (95% posterior credible interval (PCI): 0.38-0.71) and an apparent “specificity” of 99% (95% PCI: 0.98-1.00), while respiratory related conditions were detected with a “sensitivity” of 0.92 (95% PCI: 0.84-0.99) and a “specificity” of 98% (95% PCI: 0.95-1.00), when the recordings were done by meat inspectors. However, while these were reported as “sensitivity” and “specificity”, we prefer an alternative interpretation as measures of agreement between different observers,. This is because the latent class analysis used by Bonde *et al.* [8] modelled a latent condition between meat inspectors and researchers, which are likely to be correlated measurements based on common diagnostic criterion rather than independent measurements as would be expected with unrelated diagnostic tests. The latent condition is defined by the diagnostic tests (observers), and so if they do not cover “different” aspects of the condition, then the latent condition becomes what they can agree on. Consequently, accuracy estimates are not currently readily available, and in particular there are no recent data available on the correlation between findings at SHM and RMI.

The objective of this study was to compare the proportion of recorded lesions at RMI with the proportion of findings at SHM concerning the following conditions:

- RMI-Chronic pericarditis (Code 222) and SHM- Chronic Pericarditis.
- RMI-Chronic pleuritis (Code 289) and SHM-Chronic pleuritis.
- RMI-Acute/subacute pneumonia or lung necrosis, fibrinous or chronic pleuritis (Codes 258, 271, 287 and 289) and SHM-lungs with lesions.

The categories were chosen based on the actual recordings at RMI and SHM, with the above-mentioned codes specified in a government circular [2]. We hypothesised that the proportions of recorded lesions using RMI and SHM would be linearly correlated if the sampling strategy of RMI and SHM were the same for the conditions in question.

## Materials and methods

### SHM data

Post mortem examinations of the plucks (heart and lung set from each pig) as part of the SHM was done for in total 165 pig herds in the period 27 September 2011 to 29 November 2013. The data were collected as part of a project where MSD Animal Health (Ballerup, Denmark) offered veterinarians a diagnostic package to slaughter pig herds with persistent problems with respiratory diseases. This diagnostic package was offered to the largest eight veterinary pig practices in Denmark via mailed invitation letters and visits to the practices. Besides from the pathological examination, the diagnostic package included 5 blood samples from pigs in each of the age-groups 6, 9, 12, 16 and 20 weeks of age. These samples were subject to serological examinations for antibodies to *Actinobacillus pleuropneumonia* toxins (ApX I-III), swine influenza, porcine reproductive and respiratory syndrome virus and *Mycoplasma hyopneumonia* and for detection of porcine circovirus type 2 using polymerase chain reaction (PCR). The serological data were not used in this study, because the investigated age groups were different to those assessed in the present study. MSD Animal Health had no influence on the selection of herds or specific animals in the herds. Initially, the pig producer was informed to select approximately 30 “pigs representative of the herd” and mark them for SHM. However, this procedure was changed approximately half way through the project to allow slaughter house personnel to do the selection, because of logistic challenges in separating the chosen pigs from the rest of the batch. Lungs and hearts from the selected pigs were then sent to the laboratory at the Danish Pig Research Centre (Kjellerup, Denmark). At the laboratory, a gross pathological examination was carried out by one of three trained pathologists. For each herd, the proportion of the batch with different lesions was then used for further analyses. The recorded changes were the proportion of pigs with: i) Chronic pericarditis; ii) Chronic pleuritis; and iii) Lesions in the lungs.

### RMI data

From the same 165 herds, RMI data from the whole batch of pigs, which was slaughtered at the corresponding date, were obtained from the meat inspection database through the Danish Veterinary Food Administration (Glostrup, Denmark). The meat inspection codes most closely resembling the SHM diagnoses were then selected among the codes given in the government circular on meat inspection in Denmark [2] as shown in Table 1. The pigs were slaughtered at 10 different abattoirs, with 65 batches slaughtered at two abattoirs using visual meat inspection without incision in the heart, and 100 batches slaughtered at eight abattoirs using traditional meat inspection, where incision into the heart is part of the meat inspection procedures.

**Table 1:** **Corresponding categories used for disease recordings at extended meat inspection (SHM) and meat inspection codes at routine meat inspection (RMI)**

**Objective**	**SHM**	**RMI**
a)	i) Chronic pericarditis	I) Code 222: Chronic pericarditis
b)	ii) Chronic pleuritis	II) Code 289: Chronic fibrous pleuritis
c)	iii) Lungs with lesions	III) Code 258: Acute/subacute pneumonia with lung necrosis;
		Code 271: Chronic pneumonia or lung abscesses (aerogenic);
		Code 287: Fibrinous, sero-fibrinous, suppurative or putrid pleuritis (acute pleuritis);
		Code 289: Chronic fibrous pleuritis

For each parameter (see Table 1), the proportion of the batch with a positive recording of the code in question was estimated, except for the combination of Codes 258, 271, 287 and 289, where just one had to be positive for lungs with lesions deemed to be present.

### Statistical comparisons

The resulting proportions from the two separate RMI codes (Codes 222 and 289) and code combinations (Codes 258, 271, 287 and 289 combined) were compared to the proportions from the SHM as listed in the objectives and Table 1. The prevalence distributions were plotted using histograms, and the paired proportions were plotted in scatterplots. The coefficients of determination (R^2^) were estimated based on the linear relation between the proportions using the lm() function in R [9]. Although the binomial nature of the data suggests the use of a generalised linear model, we chose to use a linear model because the primary purpose of the study was to estimate the correlation between the observed prevalences rather than to fit an explanatory model with either observed prevalence as an outcome. A linear model is justified under the assumption that observed proportions from populations with similar prevalence should be highly related, irrespective of recording method, assuming that the two prevalence estimates are subject to similar sampling biases.

The analysis for pericarditis recordings was repeated using “abattoir inspection type” as a factor in the analyses. “Abattoir inspection type” was based on abattoirs that did or did not use heart incisions in addition to visual inspection. This was done due to a suspicion raised by the laboratory that hearts with positive recordings from batches with recorded Code 222 (Chronic pericarditis) would be retained at the abattoir. These sampling procedures at the abattoirs could not be confirmed due to the nature of the data, so an analysis was performed excluding batches where the prevalence of pericarditis at SHM was 0. The meat inspection and laboratory personnel were not aware that the present investigation would be initiated and were blinded to the results of any other examinations.

## Results

**Figure 1:**
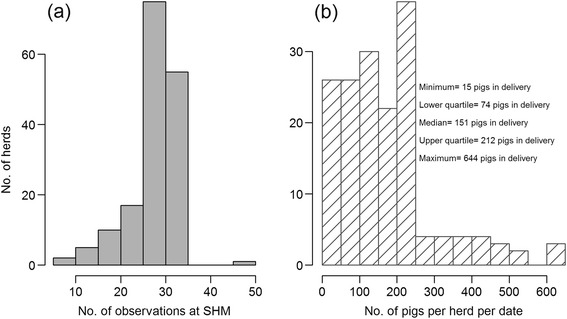
**Distribution of no. of samples per herd per date based on the (a) SHM data and (b) RMI data.**

**Figure 2:**
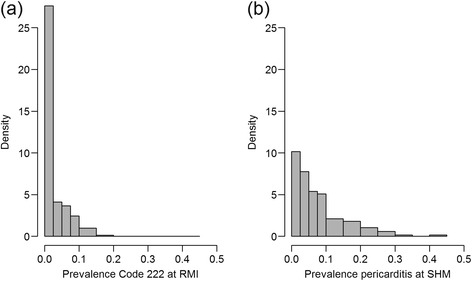
**Prevalence distributions of meat inspection Code 222 at RMI (a) and chronic pericarditis at SHM (b).**

**Figure 3:**
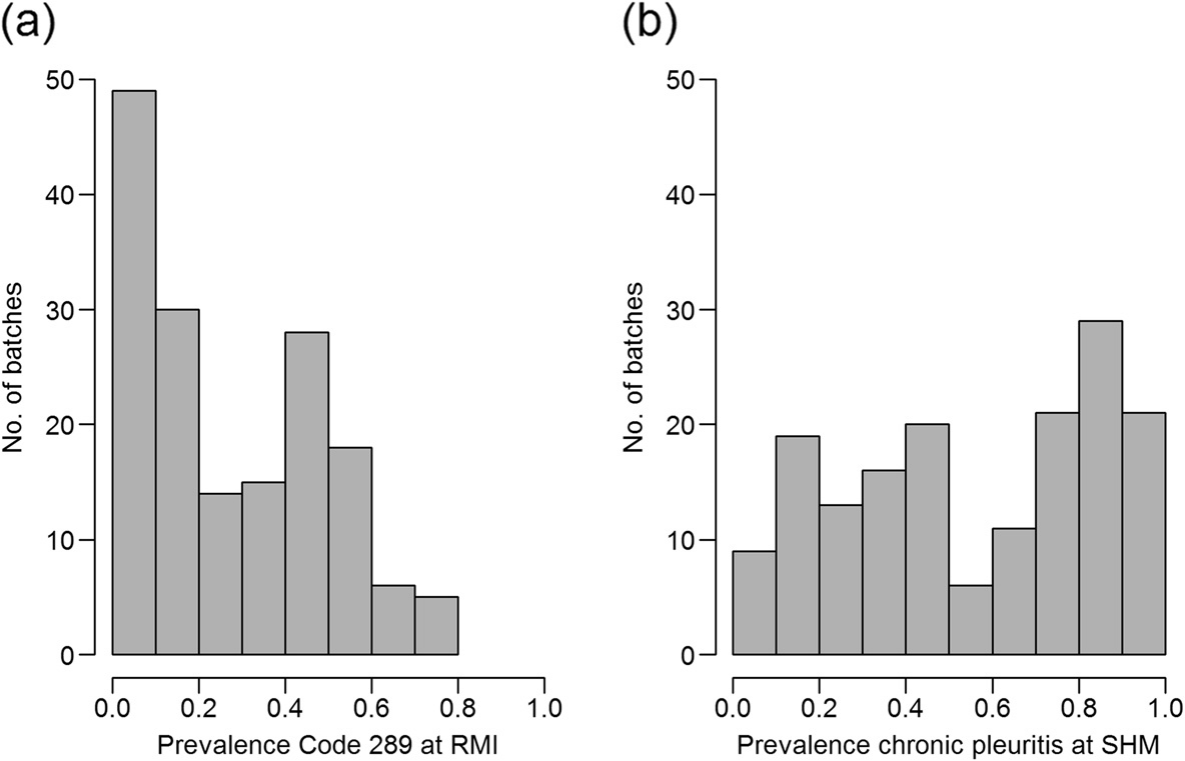
**Prevalence distributions of meat inspection Code 289 at RMI (a) and chronic pleuritis at SHM (b).**

**Figure 4:**
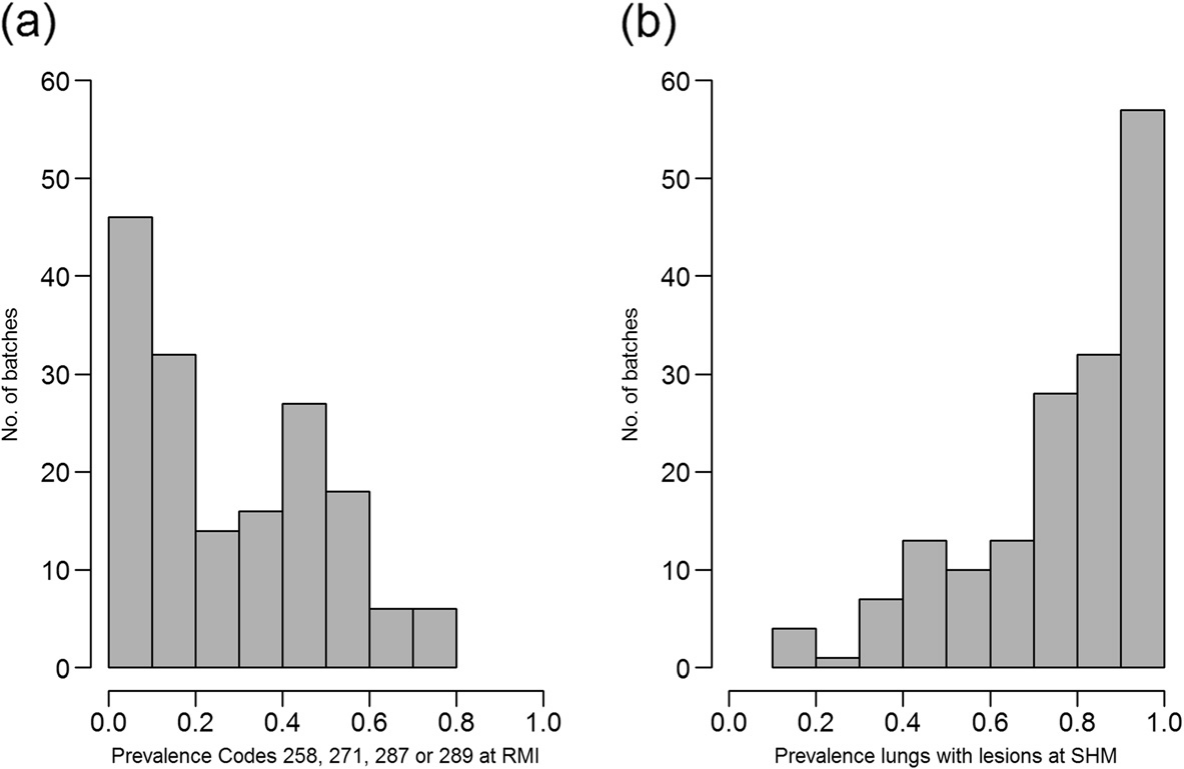
**Prevalence distributions of meat inspection Codes 258, 271, 287 and 289 at RMI combined (a) and lungs with lesions at SHM (b).**

**Figure 5:**
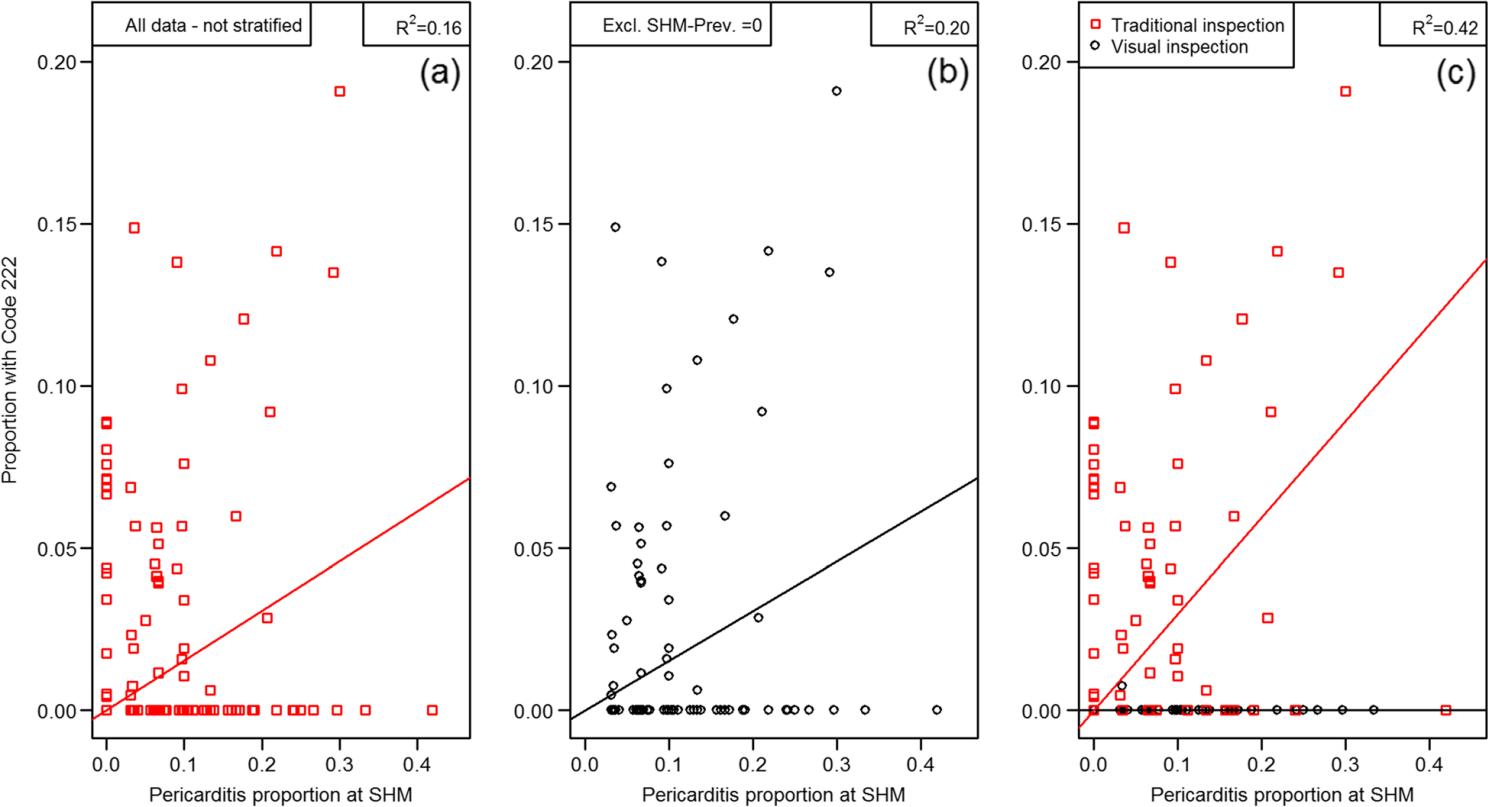
**Resulting scatterplots and adjusted R** ^**2**^ **-values for correlation between pericarditis at RMI versus SHM, based on all data (a), excluding batches where the prevalence was 0 at SHM (b) and all data correcting for the effect of using traditional or visual meat inspection, including or excluding incisions in the hearts (c).**

**Figure 6:**
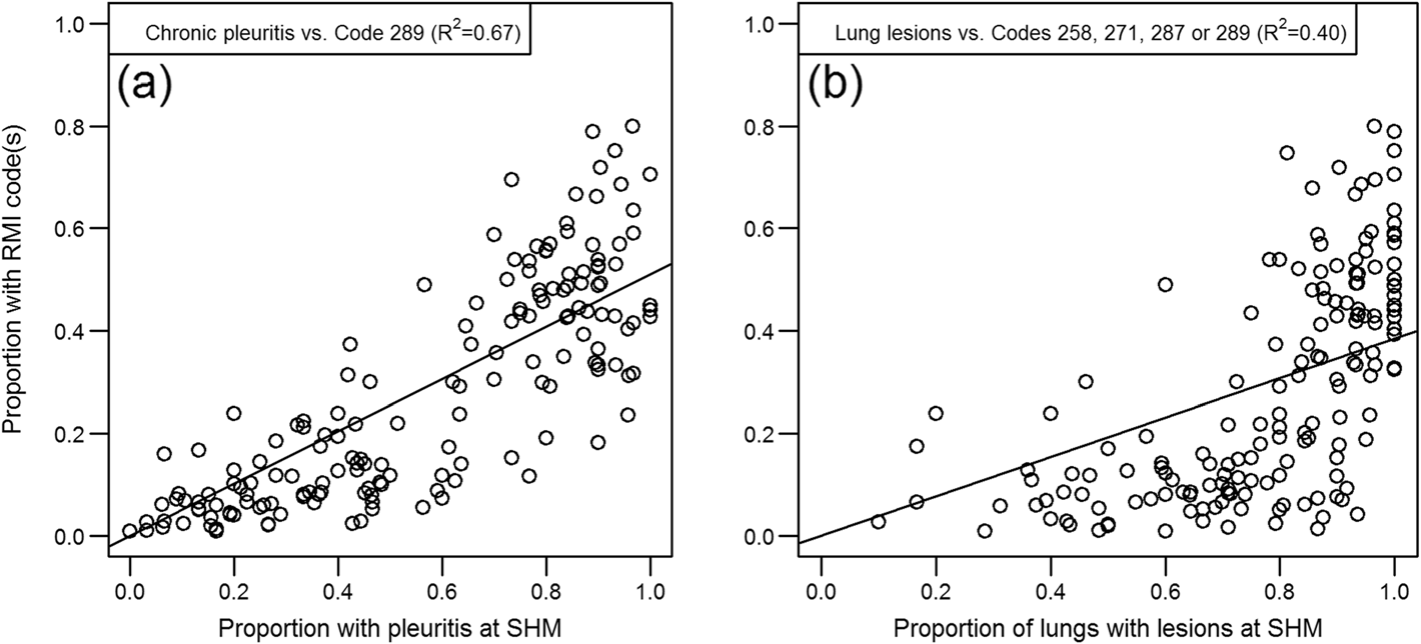
**Scatterplots and adjusted R** ^**2**^ **-values showing the correlation between RMI and SHM recordings for pleuritis and related lung conditions.**

The distribution of number of lungs from each herd on a given date as well as the number of pigs in the batch is shown in Figure 1. All but four herds submitted whole sets of plucks while three herds missed one heart, and one herd missed two hearts. The prevalence for the codes in the different pairs of proportions for Objectives a) chronic pericarditis, b) chronic pleuritis and c) lungs with changes are shown in Figures 2, 3 and 4 respectively. The overall R^2^ for the pericarditis recordings was 0.16, which improved slightly to R^2^ = 0.20 when excluding 34 complete batches of SHM recordings with a pericarditis prevalence of 0 (Figure 5a + b). Only one of the 65 batches from abattoirs using visual inspection without incisions into the heart had any chronic pericarditis recordings at RMI. The corresponding SHM prevalences for these 65 batches ranged from 0 to 33%, with 14 batches having a prevalence of 0 at SHM. Thus, when including abattoir type as a factor in the regression analysis (visual or traditional), the R^2^ improved to 0.42 (Figure 5c). The results of the correlation estimates for chronic pleuritis and lungs with lesions are illustrated and presented in Figure 6. The coefficient of determination (R^2^) between RMI Code 289 and chronic pleuritis was 0.67. There was extremely weak correlation between the combined RMI lung lesion codes (258, 271, 287 or 289) and recording of lungs with lesions at SHM, with an R^2^ of 0.40 (Figure 6).

## Discussion

This study demonstrated a substantial difference in recordings made at RMI and recordings at SHM for common conditions in pigs at slaughter, including chronic pleuritis, lungs with lesions and chronic pericarditis. The correlation between chronic pleuritis at RMI and SHM was reasonable (R^2^ = 0.67), although far from perfect. This correlation estimate suggests that some conditions at least tend to be recorded when they occur, i.e. the sensitivity of RMI compared to SHM is likely in the range that has previously been reported from Denmark [10]. However, the imperfect correlation questions the use of these results for purposes other than the original purpose of food safety. The differences can be due to different recording sensitivities due to time and observer issues. The time available at the abattoir for observing each pig or set of plucks is very limited in contrast to the time available at the laboratory. Likewise, the abattoir technicians at the abattoir have a different educational background than the trained pathologists, who may be more observant to details. Consequently, the use of RMI recordings for anything other than originally intended may not be the most reasonable and the results provided here may merely serve as a warning against such comparisons. It should be noted though, that the present study did not address if the RMI recordings were adequate for the purpose they are intended for.

If we instead assess chronic pericarditis in greater detail, the sources of variation may become more evident. Abattoirs using visual meat inspection reported very few cases of Code 222, despite high proportions of pericarditis at SHM for the same batches (Figure 5c). Therefore, in addition to lack of recording sensitivity at traditional meat inspection [7], a large proportion of chronic pericarditis cases may not be recorded at visual inspection, resulting in a further loss of overall sensitivity.

The current study has a number of shortcomings. Firstly, sampling of herds was not done at random, and herds with many lungs with lesions may have been more likely to be included as a result of the selection procedure. While this may well introduce a bias in the estimated prevalence of the recordings, the correlation estimates should not be affected if all cases are recorded with similar sensitivity. A more important limitation is the sampling of plucks at the abattoir. Sampling was originally intended to include “normal” pigs selected by the farmer, but was later on replaced by selection performed by abattoir technicians. This was done because the abattoir technicians were unable to identify the pigs preselected by farmers, and consequently chose pigs based on convenience. Had farms been included based on higher occurrence of specific diseases this would increase the prevalence in both the RMI and the SHM material, but this would not be expected to have any impact on the linear relationship. There was no reason to believe that the plucks were selected based on disease occurrence except in those situations where no pericarditis cases were found at SHM. This may be the result of some abattoirs not shipping plucks with affected hearts to SHM, which is possible for 15 of the 165 batches in our dataset (Figure 5). For practical purposes, sampling was therefore considered random, and consequently selection bias at organ level was not believed to have any relevant impact on the results. This is supported by exclusion of the zero-prevalence SHM pericarditis data, which had a limited effect on the results (Figure 5a + b). Furthermore, the reasonable correlation found for chronic pleuritis suggested that sampling was not considerably affected by selection bias at the abattoir.

The study was conducted as a retrospective study, which was advantageous in that blinding was easier and none of the technical staff were aware of the investigation. However, the lack of information at the level of individual carcasses in both RMI and SHM meant that we were not able to calculate sensitivity or specificity estimates. These estimates would probably also have been biased, because blinding would be logistically challenging in a study, where all study objects need to be carefully marked for further studies.

Another more important feature may be the difference in the conditions recorded at RMI and SHM. The similar condition of chronic pleuritis and chronic fibrous pleuritis were compared, but they are not necessarily completely identical. The SHM code chronic pleuritis is recorded based upon findings on the pleura of the lung alone, whereas the RMI code 289 is recorded based upon findings on the pleura of the lung as well as the pleura of the intrathoracic cavity. However, the expected higher sensitivity of the SHM recordings might level out the eventual difference. The most prominent difference would probably be with “lungs with lesions” at SHM vs. the four abattoir codes 258, 271, 287 or 289. The SHM recording would likely include mycoplasma-like lesions, which commonly results in lesions in Danish pigs, but these lesions may rarely be recorded at slaughter. Mycoplasma associated lesions may be very relevant to herd health advisors and consequently also relevant at SHM, but less so for food safety reasons and therefore of limited relevance at RMI. This suspected lack of recordings at RMI regarding mycoplasma associated lesions would explain some of the differences related to lesions of the lungs. However, the correlation between these recordings still reach a moderate level (R^2^ = 0.40), but this is highly due to code 289 being included.

A linear model was used and should be the most appropriate, given the premise that the conditions should be similar and if the purpose is the same, then the recording prevalence should also be, irrespective if SHM or RMI is carried out. However, further inspection of the scatter plots in Figure 5 suggests that the relations are more likely quadratic or cubic than linear, and if we assess the relation including quadratic and cubic terms, such models actually provide better model fit (based on Akaike’s Information Criterion) and R^2^ changed from 0.67 to 0.89 for chronic pleuritis (data not shown). It can be speculated if the cubic relationship is indeed due to a practice at the abattoirs where if one pleuritis is recorded in a batch, then more lesions are likely to be recorded in that batch, irrespective of the true prevalence.

If this is indeed the case, it again highlights that the two data sources are collected for different purposes, which may be the primary reason for the difference in the results. However, it also highlights the need for a specific purpose given for each lesion recording. If a specific purpose is not given, the quality of the recording may drop. For example, the reasons for recording pericarditis at SHM and at RMI may be completely different, and there appears to be less interest in detection of chronic pericarditis in modern meat inspection, resulting in fewer cases of pericarditis at RMI at abattoirs using visual inspection compared to those using traditional inspection with incisions to the hearts. The numbers reported from the present study do not differ greatly from the national Danish RMI data from 2012. In these data, approximately 10 million pigs were slaughtered at the nine biggest pig abattoirs using incisions to the heart and 7.3 million pigs were slaughtered at the two abattoirs using visual inspection. The former had a prevalence of recorded chronic pericarditis of 3.1% (around 308,491 cases), whereas the latter had a recording prevalence of 0.019% (approximately 1,385 pigs) (unpublished data from The Veterinary Food Administration, Glostrup, Denmark). In a Danish release assessment, it was concluded that visual meat inspection with lack of incisions to the heart would not affect the detection of conditions on the outside of the heart (e.g. chronic pericarditis), because the hearts are still observed visually [11]. However, the prevalence estimates suggest differently, and the results of the present study support this notion. Lastly, this study pertains to a limited number of organs and recordings, which is a potential limitation to a more general interpretation of our findings. However, despite the potential limitations in terms of data quality in the present study, our results are consistent with the findings of others (Enøe *et al.*, 2003; meat inspection data from 2012 mentioned above), and we believe the overall conclusions of our results to be valid.

## Conclusion

This study demonstrated important discrepancies between the findings of RMI and SHM, with variable correlation in reported prevalence for different conditions. There was a reasonable correlation between the apparent prevalence of chronic pleuritis and lungs with lesions in pigs recorded at RMI compared to SHM, but the correlation was poor for chronic pericarditis. The latter can partly be explained by abattoirs failing to detect chronic pericarditis when conducting visual meat inspection without incisions to the heart. We therefore recommend caution when using data from meat inspection for purposes unrelated to the food-safety purpose for which this data was originally intended.
